# There and back again: historical biogeography of neotropical magnolias based on high-throughput sequencing

**DOI:** 10.1186/s12862-025-02379-7

**Published:** 2025-04-30

**Authors:** Salvador Guzman-Diaz, Fabián Augusto Aldaba Núñez, Emily Veltjen, Pieter Asselman, José Esteban Jiménez, Jorge Valdés Sánchez, Guillermo Pino Infante, Ricardo Callejas Posada, José Antonio Vázquez García, Isabel Larridon, Suhyeon Park, Sangtae Kim, Esteban Manuel Martínez Salas, Marie-Stéphanie Samain

**Affiliations:** 1 Red de Diversidad Biológica del Occidente Mexicano, Instituto de Ecología, A.C, Pátzcuaro, Michoacán Mexico; 2 Ghent University Botanical Garden, Ghent University, Gent, Belgium; 3 Department of Biology, Systematic and Evolutionary Botany Lab, Ghent University, Gent, Belgium; 4 Herbario Luis A. Fournier Origgi, Centro de Investigación en Biodiversidad y Ecología Tropical (CIBET), Universidad de Costa Rica, San Pedro de Montes de Oca, San José, Costa Rica; 5 Department of Biology, Florida Museum of Natural History and University of Florida Herbarium, University of Florida, Gainesville, Florida USA; 6 Herbario PMA, Facultad de Ciencias Naturales, Exactas y Tecnología, Universidad de Panamá, Panama City, Panama; 7 Museo de Historia Natural, Universidad Nacional Mayor de San Marcos, Jesús María, Lima, Peru; 8 Grupo de Biotecnología, Laboratorio de Taxonomía de Plantas Vasculares, Instituto de Biología, Universidad de Antioquia, Medellín, Colombia; 9 Herbario IBUG, Instituto de Botánica, Departamento de Botánica y Zoología, Centro Universitario de Ciencias Biológicas y Agropecuarias, Universidad de Guadalajara, Nextipac, Zapopan, Jalisco Mexico; 10 Royal Botanic Gardens, Kew, Surrey, UK; 11 Department of Biology, Sungshin Women’s University, Seoul, Korea; 12 Departamento de Botánica, Herbario Nacional de México, Instituto de Biología, Universidad Nacional Autónoma de México, Mexico City, Mexico

**Keywords:** Biogeography, Divergence times, Magnoliaceae, Neotropics, Phylogenomic, Target sequencing

## Abstract

**Background:**

The Neotropics are considered one of the most biodiverse areas in the world, housing at least one third of all vascular plant species. One of the genera that has diversified in the Neotropics is *Magnolia*, with about 174 species of three sections (*Macrophylla*, *Magnolia* and *Talauma*) endemic to the Americas. In this work, we study the biogeographic history of the Neotropical *Magnolia* species using high-throughput sequencing data. Sequences from 39 species (38 from *Magnolia* and one from the sister genus *Liriodendron*) were assembled. The dataset contained sequences from 239 nuclear targets and complete chloroplast genomes. Phylogenomic hypotheses and the ancestral distribution range of *Magnolia* were reconstructed.

**Results:**

The results of the calibrated phylogenetic hypotheses and ancestral range construction suggest that the earliest arrival in the Neotropics were the ancestors of section *Talauma* (38 million years ago), which colonized the Pacific region. This early presence in South America suggests long-distance, overwater dispersal from North America, the presumed origin of the genus *Magnolia*. The analysis and the extant *Talauma* distribution indicate a south to north recolonization. The ancestors of the other two Neotropical sections, *Magnolia* and *Macrophylla*, migrated around 19 mya from Asia to North America, radiating southward to the Neotropics afterwards, around 11 mya.

**Conclusions:**

Our results suggest that Neotropical magnolias originated from a North American ancestor. The current sections arrived at the region independently influenced by climatic processes such as temperature drops or the Miocene Climatic Optimum. Additionally, geological processes, such as the movement of the South and North American land masses and the emergence of the Panama isthmus, facilitated the migration between continents.

**Supplementary Information:**

The online version contains supplementary material available at 10.1186/s12862-025-02379-7.

## Background

The Neotropics are considered one of the most biodiverse areas of the world, especially for plants [1, 2], as at least one-third of all known vascular plant species occur there [3]. This biogeographical region extends from central Mexico up to southern Brazil [4], and includes some of the most important biodiversity hotspots [5]. Different reasons have been proposed to explain the high diversity of the Neotropics, such as the large area, high environmental and climatic heterogeneity, and the evolutionary history of the clades that inhabit the region [6, 7]. The latter is of special biogeographic interest for the current angiosperm biodiversity of the Neotropics because many species are descendants of ancient lineages that originated in different parts of the world [8–10].

Some of the angiosperm groups that constitute the extant Neotropical flora are shared with northern regions such as Asia and North America, including boreotropical families such as: Burseraceae, Fagaceae, and Magnoliaceae [11, 12]. The boreotropical flora consists of several angiosperm lineages which inhabited the boreal territories of Laurasia during the late Cretaceous and migrated to southern latitudes in later periods [12, 13]. This flora was originally distributed on the land masses that currently constitute North America and Europe. After the separation of the North American and European plates, migration between the continents persisted through the North Atlantic Land Bridge. This connection continued intermittently until the late Eocene [14, 15]. At the same time the migration across the Bering strait, connecting Eastern Asia and North America, began to increase, promoted by the then smaller distance between North America and Eurasia [11, 16, 17]. This allowed for an interchange between elements from both continents, and this exchange produced a complex biogeographic pattern, such as the disjunct distribution present in angiosperm families across different continents [12].

Other families belonging to the current Neotropical flora (e.g., Annonaceae, Arecaceae, Euphorbiaceae, Myristicaceae, Winteraceae) started their evolution in ancient Gondwana [17, 18]. About 100 million years ago (mya), during the Cretaceous, this supercontinent started to divide in current Africa and South America [6]. As a consequence, the latter has remained isolated for almost 70 million years, resulting in a significant diversification of clades[6]. At about 34 mya, many plant groups began to migrate between South and North America [6]; although Central America remained submerged at that time, those migrations could have occurred by the emergence of the Greater Antilles and Aves Ridge (GAARlandia) or by long distance dispersal [6, 19, 20]. All these processes were influenced by different climatic changes that occurred simultaneously, such as the Eocene/Oligocene transition that happened about 34 mya, causing a global mean temperature drop of about 4 °C and a massive increase of the Antarctic ice sheets [21]. These changes promoted the colonization of new territories by several plant groups [12], and this interchange of elements between North and South America began to increase with the emergence of the Panama land bridge. The gap between these two continents began to close about 25 - 23 mya and both land masses continued their approximation until the complete emergence of the Panama Isthmus about 3 mya [6, 12, 17]. However, different periods of increased interchange occurred in the latest 30 mya [22].

The biogeography of the Neotropical flora is also influenced by environmental and climatic heterogeneity of the region [6, 23]. It has been observed that there is an important relationship between species diversity and the topographic and environmental heterogeneity created by mountain ranges [24]. In the Neotropics there are a series of mountain ranges that run almost continuously across the entire region, from northern Mexico up to Argentina, being the product of diverse periods of tectonic and volcanic activity [23, 25, 26]. These mountain ranges facilitated the migration of species from more temperate climates to tropical regions [2, 27], while creating new ecosystems and favoring the diversification of some of these groups [23, 28–31]. The emergence of mountain ranges also created barriers that could favor the diversification by vicariance of other plant groups that inhabited the surrounding lowlands [29, 32, 33].

Magnoliaceae is one of the earliest diverging families of the angiosperms and the Neotropics are one of their main centers of diversity. This family comprises two genera: *Liriodendron* L. and *Magnolia* L.*,* and includes about 358 [34] species of trees and shrubs. About 172 species inhabit in the Neotropics belonging to three sections: *Macrophylla*, *Magnolia* and *Talauma* [35]. The rest of the species inhabit temperate and tropical regions of North America, eastern and southeastern Asia [34–38].

Different estimated ages have been proposed for the Magnoliaceae and their Neotropical clades. Estimations are based on fossil evidence, which are then used for further estimates using calibrated phylogenetic hypotheses. Fossils assigned to this family have been found in North America, Greenland and Europe, with an estimated age of approximately 100 million years, suggesting a boreotropical origin [12, 39–41]. Calibrated phylogenetic hypotheses have been proposed for the family based on different sets of taxa, genetic data, and calibration schemes. Nie et al. [42] used three nuclear genes to estimate the divergence times of 86 Magnoliaceae taxa. They suggested an estimated 54 mya for the subfamily Magnolioideae and between 30 and 47 mya for the Neotropical sections *Magnolia* and *Talauma*. A similar pattern was found by Dong et al. [43]who calculated the divergence times of 48 Magnoliaceae species using a combination of nuclear and plastid markers. Veltjen et al. [44] analyzed 62 Magnoliaceae taxa using a combination of eleven markers (five nuclear and six from the chloroplast). This work focused on Neotropical and Caribbean taxa and found divergence estimates for the three Neotropical sections: 30 mya for section *Talauma*, 4.8 mya for *Macrophylla* and 9.3 mya for *Magnolia*.

Although previous studies improved the knowledge on the evolution of the genus, the main biogeographic factors that influenced the distribution of the family in the Americas remain unknown. About half of the Magnoliaceae species diversity inhabits the Americas but only a few have been included in previous studies [13, 38, 42]. Recent works have addressed the evolution and biogeography of Caribbean Magnolias [44, 45]. These studies demonstrated that *Magnolia* arrived in the Caribbean in four different events: 1) the arrival of subsection *Cubenses* in Cuba, 2) the entrance of section *Talauma* in Cuba, 3) the arrival of *M. dodecapetala* in the Lesser Antilles and 4) the colonization event of *M. virginiana* from North America to Cuba. However, the evolution of the species from the rest of the Neotropics is still unclear.

In recent years, there have been significant advances in sequencing with the development of the so-called High Throughput Sequencing techniques (HTS). These techniques enable researchers to access to huge quantities of genomic data at low cost [46]. These strategies allow to resolve phylogenomic questions in different groups of angiosperms, such as the phylogenomic relationship of the species or the biogeographic history of some families [47, 48]. For the case of Magnoliaceae, a specific bait set for HTS that includes 490 nuclear markers has recently been developed (Kim *et al.*, in press). Customized sets could be extremely useful to perform successfully HTS studies in some complex angiosperm groups [49].

In this study we aim to study the biogeographic history of the Neotropical *Magnolia* species using HTS data. Our objectives for this study were to: 1) Reconstruct a phylogenetic hypothesis of the Neotropical representatives of the genus *Magnolia* using genomic chloroplast and nuclear data; 2) identify the main colonization routes followed by the ancestors of current *Magnolia* taxa in the colonization of the Neotropics based on a combination of the assembled genomic data; and 3) estimate the age of the Neotropical clades of *Magnolia* the probable driving factors of their origin and radiation.

## Materials and methods

### Sampling, DNA extraction, sequencing and assembly

**Fig. 1:**
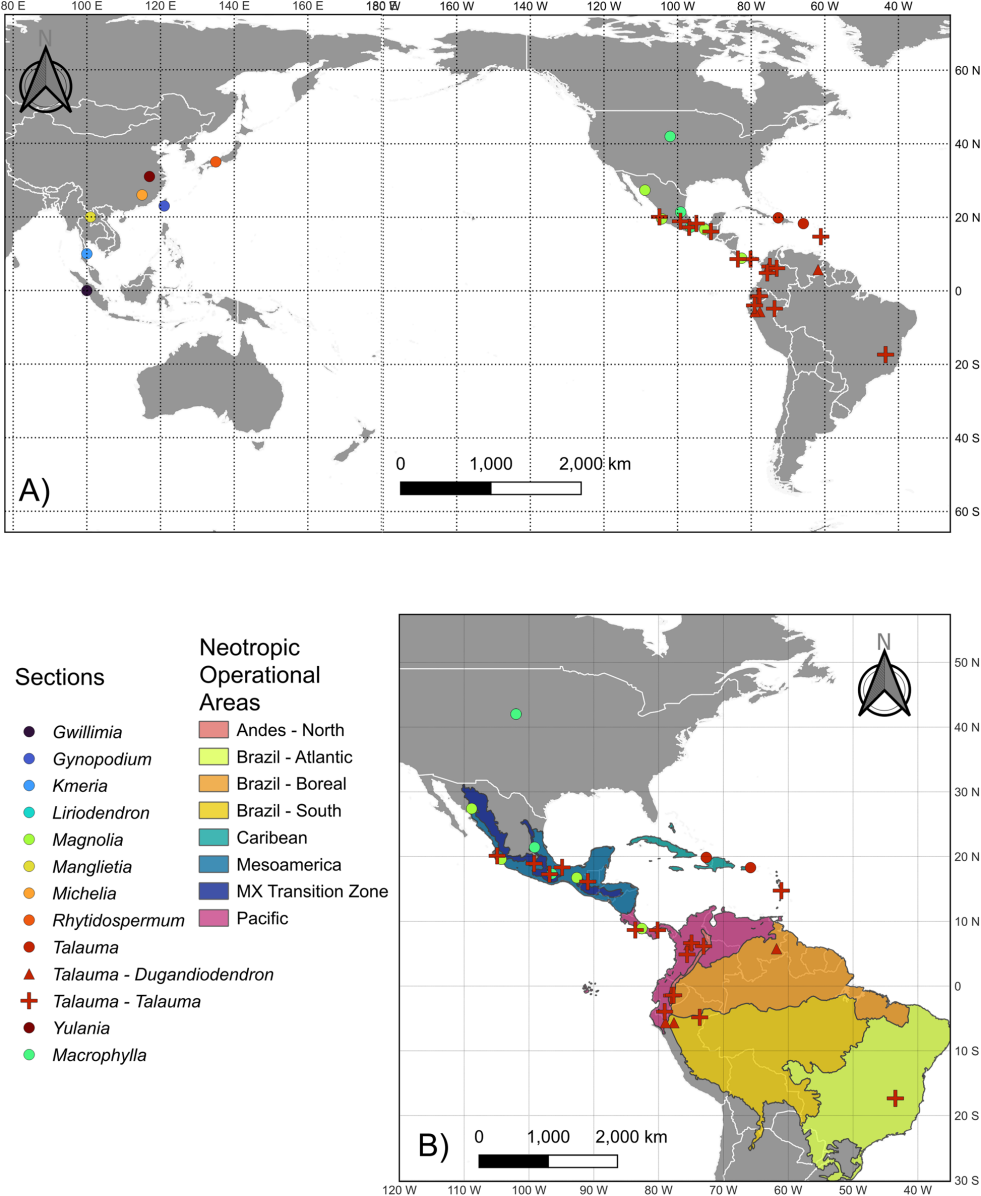
**A** Localities of the 39 Magnoliaceae species selected from the whole distribution area of the genus. **B** Localities of the 29 Neotropical species included in the analysis and the subregion delimited for this study. Markers indicate the section to which each species belongs. Colors in **B**) represent the operational areas defined for the ancestral range reconstruction based on the biogeographical provinces of Morrone and Löwenberg-Neto. In addition to the Neotropical areas, the Nearctic region and Asia were also considered as operational areas for the analysis. This figure is an original creation and does not derive from any other source

Field work was carried out in Colombia, Costa Rica, Ecuador, Mexico, Panama, Peru and Puerto Rico between 2015 and 2019. Young leaves were collected and dried in silica gel. Voucher specimens were deposited in local herbaria as well as in the herbaria GENT, IEB and/or MEXU [50] (Table 1); sampling was completed with herbarium material and collections from botanical gardens when necessary. Fourteen herbaria were consulted in this study: Sungshin Women's University (SWU), Seoul National University (NPRI), Instituto de Ecología, A.C. (XAL), Missouri Botanical Garden (MO), Jepson Herbarium (JEPS), Swedish Museum of Natural History (S), Universidad de Antioquia (HUA), Centro Regional del Bajío (IEB), Royal Botanic Gardens (K), Universidad de Guadalajara (IBUG), Universidad Nacional Mayor de San Marcos (USM), Universidad de Costa Rica (USJ). Determination of plant material was carried out by the authors to ensure a correct identification of each species. A total of 39 samples were included in the analysis; of which, twenty-nine samples corresponded to Neotropical *Magnolia* species. These represent the complete Neotropical distribution of the genus (Fig. 1), as well as all the sections and subsections that inhabit the region (Table 1). Additionally, six Asian and three Nearctic species of *Magnolia* were included. Finally, one species of *Liriodendron*, the sister genus of *Magnolia*, was used as outgroup [38].

**Table 1:** Thirty-nine Magnoliaceae species included in the analysis

Genus	Section	Subsection	Species	Locality	Voucher (herbarium)
*Magnolia*	*Gwillimia*	*Gwillimia*	*M. henryi*	South China Botanical Garden, China	Kim 2017 - 0216 (SWU)
	*Gynopodium*	*Gynopodium*	*M. kachirachirai*	Kunming Botanical Garden, China	Kim 2014 - 0830 (SWU)
	*Kmeria*	*Kmeria*	*M. septentrionalis*	South China Botanical Garden, China	Kim 1053 (NPRI)
	*Macrophylla*	*NA*	***M. dealbata***	Chungcheongnam-do (Chollipo arboretum), South Korea	Kim 1008 (NPRI)
			*M. grandiflora*	Florida, USA	Kim 2019 - 0083 (SWU)
			*M. macrophylla*	Chollipo Arboretum, Rep. of Korea	Kim 1015 (NPRI)
			***M. rzedowskiana***	San Luis Potosí, Mexico	Mata 1118 (XAL)
			***M. vovidesii***	Jalisco, Mexico	Kim 2019 - 070 (SWU)
	*Magnolia*	*NA*	***M. iltisiana***	Jalisco, Mexico	Kim 2019 - 084 (SWU)
			***M. panamensis***	Chiriquí, Panama	McPherson 15882 (MO)
			***M. sharpii***	Chiapas, Mexico	Collection #80.0066 (JEPS)
	*Manglietia*	*Manglietia*	*M. grandis*	Kunming Botanical Garden, China	Kim 2015 - 0031 (SWU)
	*Michelia*	*Michelia*	*M. foveolata*	Kunming Botanical Garden, China	Kim 2015 - 0021 (SWU)
	*Rytidospermum*	*Rytidospermum*	***M. obovata***	Chollipo Arboretum, Rep. of Korea	Kim 1046 (NPRI)
	***Talauma***	***Cubenses***	***M. emarginata***	Centre, Haiti	Ekman 4339 (S)
			***M. splendens***	Puerto Rico	Kim 1108 (NPRI)
		***Dugandiodendron***	***M. bankardiorum***	Zamora, Ecuador	Kim 2015 - 0114 (SWU)
			***M. coronata***	Antioquia, Colombia	Veltjen et al. 2019 - 016 (HUA)
			***M. jaenensis***	Cajamarca, Peru	Samain et al. 2018 - 001 (IEB)
			***M. ptaritepuiana***	Bolivar, Venezuela	Steyermark 59547 (K)
		***Talauma***	***M. allenii***	Coclé, Panama	Samain et al. 2019 - 016 (IEB)
			***M. dodecapetala***	Magnolia Grove Arboretum, USA	Kim 1106 (NPRI)
			***M. kichuana***	Ecuador	Kim 2015 - 0112 (SWU)
			***M. lacandonica***	Chiapas, Mexico	2017 - 009 (IEB)
			***M. macrocarpa***	Oaxaca, Mexico	Dominguez & Rodríguez (IBUG)
			***M. mexicana***	Morelos, Mexico	Samain & Martínez 2020 - 004 (IEB)
			***M. ofeliae***	Jalisco, Mexico	Vázquez-García & Muñiz-Castro 8979 (IBUG)
			***M. ovata***	Brasil	Prance & Silva (K)
			***M. pastazaensis***	Patuca, Ecuador	Kim 2015 - 0113 (SWU)
			***M. rimachii***	Loreto, Peru	Flores 2120 (USM)
			***M. silvioi***	Antioquia, Colombia	Veltjen et al. 2019 - 017 (HUA)
			***M. virolinensis***	Virolin, Colombia	Serna 2639 (HUA)
			***M. wetteri***	Puntarenas, Costa Rica	Jiménez 4606 (USJ)
			***M. wolfii***	Pereira, Colombia	Veltjen et al. 2019 - 002 (HUA)
			***M. zamorana***	Zamora Chinchipe, Ecuador	Kim 2015 - 0115 (SWU)
			***M. zoquepopolucae***	Veracruz, Mexico	Aldaba 247 (IEB)
	*Yulania*	*Tulipastrum*	*M. acuminata*	Chollipo Arboretum, Rep. of Korea	Kim 1001 (NPRI)
		*Yulania*	*M. biondii*	Chollipo Arboretum, Rep. of Korea	Kim 1003 (NPRI)
*Liriodendron*			*L. tulipifera*	Chollipo Arboretum, Rep. of Korea	Kim 1044 (NPRI)

Neotropical species and sections, subsections and species are in **bold**. Species are ordered by section, subsection and species name. Classification follows the one proposed by [36]

Extraction of DNA was performed using a modified CTAB protocol [51]. DNA quality was assessed using a spectrophotometer (Nanodrop 2000 UV-Vis). All samples were analyzed using a HTS approach with two different strategies: the first one consisted of a nuclear dataset based on a Target Capture Sequencing (TCS). To this end, the nuclear bait set developed by Kim et al. (in press) was used, which includes 490 nuclear markers produced for the Magnoliaceae family (File S4). The second one consists of a chloroplast dataset based on a Whole Genome Sequencing (WGS). In both cases library preparation and sequencing was performed by Rapid Genomics (Gainesville, Florida, USA) following a HiSeq protocol on an Illumina platform. Paired end reads of 150 bp where produced.

Demultiplexing was carried out using BCLtofastq [52]. A first quality check was performed using FastQC v. 0.11.7 [53] and multiQC [54]. Trimmomatic v. 0.38 [55] was used to filter low-quality reads and perform an adapter trimming. A sliding window of 5:20 was applied and reads of less than 30 bp length were removed. A second quality check with FastQC and multiQC was performed to ensure correct adapter removal. The assembly of the chloroplast dataset was carried out using the pipeline GetOrganelle [56]; this uses Bowtie2 [57], SPAdes [58] and BLAST [59] to assemble a complete chloroplast sequence. As seeds for the assembly, complete chloroplast sequences from three Neotropical *Magnolia* species (*M. pacifica* subsp. *tarahumara* A. Vázquez/MN990636.1, *M. dealbata* Zucc./NC_023235.1, and *M. ovata* (A. St. Hil.) Spreng./NC_048993.1) were selected and downloaded from the NCBI GenBank database [60]. The nuclear dataset assembly was performed using the pipeline HybPiper [61]; which uses BWA [62], biopython [63] and SPAdes to map reads against a series of targets and extract the sequences of exons, introns or both (supercontig) for each locus. Exons were used in subsequent analysis. Targets with paralog warnings, as detected by HybPiper, were removed from the dataset. The nuclear set developed by Kim et al. (in press) for the Magnoliaceae family was used as target for the assembly.

### Phylogenetic analysis and fossil calibration

Previous results showed that the chloroplast and nuclear data produce different topologies in *Magnolia* [35]. Nevertheless, these differences are minimal and primarily concern the arrangement of *Magnolia* sections *Magnolia* and *Macrophylla*, whereas all other Neotropical clades remain unaltered. Based on this, we decided to use three different datasets: a) a complete chloroplast sequences dataset, b) a combined nuclear dataset of the 239 targets assembled and c) a combined dataset that included both the complete chloroplast sequence assembled and 239 nuclear loci shared for all the *Magnolia* species analyzed. Each of the datasets where aligned with MAFFT v. 7.508 [64] using the auto flag. For the nuclear and the combined datasets, each of the nuclear loci were considered a different partition. For the combined dataset the chloroplast genomes were also considered as a different partition in the complete sequence alignment, making a total of 240 partitions. The complete sequence alignments have lengths of 161,886 bp for the chloroplast dataset, 286,646 bp for the nuclear dataset and 448,532bp for the combined dataset. To test the phylogenetic relationships of the Magnoliaceae species included in the analysis we constructed Maximum Likelihood (ML) species trees using IQtree v.2.1.4 [65] and the three sequence alignments defined. The program was allowed to identify the substitution model for each partition of the alignment using ModelFinder [66] and performed 1000 replicates of ultrafast bootstrap [67].

To analyze the effect of different spatiotemporal processes in their evolution, we reconstructed a dated phylogenetic hypothesis. Based on the results from the ML analysis, the combined dataset was used to infer a calibrated phylogeny using Bayesian Inference with BEAST v. 2.6.7 [68]. For this the substitution model was estimated using the bModelTest v. 1.2.1 package [69]. Considering that *Magnolia* consists of several sections and subsections, a random local clock was selected to allow for changes in the substitution rates between the different clades [70]. Finally, Calibrated Yule mode was applied [71]. We calibrated our tree using two uniform priors: one for the genus and another one for the family. For the first one, the crown node of *Magnolia* was set with a lower limit of 44 mya. This was based on the *Magnolia tiffneyi* fossil [72] which dates from the mid Eocene. The upper limit was set to 70 mya based on the estimated age of the family according to [73]. Although younger, estimations for the genus have been proposed [42, 44], we opted for the oldest one to allow a greater variation. The second uniform prior was set for the family Magnoliaceae; for this, on the one hand, a maximum of 98 mya was set based on the estimated age of the *Archaeanthus* fossil [39]. This fossil has been assigned as one of the oldest members of the Magnoliaceae [40]. On the other hand, we set the minimum for the Magnoliaceae family at 44 mya following the minimum bound previously set for *Magnolia*. Based on the knowledge of the family and following the BEAST manual recommendation [74], a most recent common ancestor prior was set for each of the three Neotropical sections (*Macrophylla*, *Magnolia* and *Talauma*). This was made with the objective of improve convergence times and the stability of the software [75]. The BEAST analysis was set to run for 1000 million generations with 10 % as burn-in. The convergence values were checked periodically with Tracer v. 1.7 [76] to ensure an effective sample size (ESS) of 200. TreeAnnotator v. 2.6.7 [68] was used to create a maximum clade credibility tree with node height representing the mean heights.

### Ancestral range reconstruction

To identify the probable origin of the Neotropical *Magnolia* clades, we estimated the ancestral range of the family. The software environment for statistical computing R v. 4.2.2 [77] and the BioGeoBEARS package [78, 79] were used to estimate the ancestral areas. The delimitation of the region was based primarily on the biogeographic provinces proposed by Morrone [4, 80]. To reduce the number of areas to analyze, the original provinces were merged according to the ecological similitudes and the distribution of the *Magnolia* species. Ten Operational Areas were defined (Fig. 1B), eight for the Neotropics: Andes-North, Brazil-Atlantic, Brazil-Boreal, Brazil-South, Caribbean, Mesoamerica, Mexican Transition Zone, and Pacific. Nearctic and Asia were used as additional operational areas. The calibrated tree was used to test a total of 24 different dispersal models. Firstly, we established five time periods for all models to be tested: 0–3 mya, 3–20 mya, 20–30 mya, 30–40 mya and 40–120 mya. Six null models were defined that included three different base models (DEC, DIVALIKE and BAYAREALIKE) along with their jump dispersal (+J) variants. The null models used a dispersal matrix with equal probability of dispersal (1) among all areas. Then three different variations for the dispersal probabilities were set: 1) a “Panama” model with different dispersal probabilities defined for the period before and after the closure of the Panama isthmus (3 mya). Dispersal probabilities between adjacent regions were defined as half the probability of remainder in the same region (0.5), and the dispersal probabilities across water was half the probability of dispersal across land (0.25). For all the time periods before the closure we reduced the dispersal probabilities between Central and South America, setting the probabilities for the dispersal between Mesoamerica and Pacific regions to 0.01. 2) A “Closing Americas” model that includes three time periods: the period after the closure of the Panama isthmus following the current dispersal probabilities; the period from the closure and up to 20 mya, simulating the probabilities of overwater dispersal between Central and South America. For this, probabilities for the dispersal between the Mesoamerican and Pacific regions were set as half the current (0.5); and the period before 20 mya, where the dispersal probabilities between Central and South America were set to 0.01. 3) A “GAARLANDIA” model with five time periods: 0–3 mya, 3–20 mya, 20–30 mya, 30–40 mya and 40–120 mya; the first three models follow the closing Americas model. The fourth period (30–40 mya) presented an increased dispersal probability in the routes from and to the Caribbean region, while in the last period the dispersal probabilities are the same as in the period from 20–30 mya. For all models we defined a maximum of two regions allowed per species based on the distribution ranges of the current species. All models where evaluated based on their AICc, and their model weight(AICc_wt), representing the relative support for each model [81].

## Results

### Sequencing and assembly

From the chloroplast genome assembly of the species, we achieved a complete circular chloroplast sequence for 36 species (Table 2). For *M. emarginata* Urb. & Ekman, the assembler recovered a nearly complete chloroplast sequence which was used in successive analyses. For *M. macrophylla* Michx. and *L. tulipifera* L., we were not able to assemble a suitable sequence. For these two species accessions from NCBI GenBank were used in subsequent analyses (accessions NC_020318 and DQ899947, respectively). Chloroplast length of the assembled sequences varied from 159,188 bp in *M. ptaritepuiana* Steyerm. to 160,087 bp in *M. obovata* Thunb. Mean depth coverage varied from 32.7 × in *M. ovata* (A.St.-Hil.) Spreng. To 3586.13 × in *M. sharpii* Miranda.

**Table 2:** Sequencing and assembly results of the chloroplast genomes and nuclear targets from the 39 Magnoliaceae species

					Chloroplast assembly			Nuclear loci assembly	
Genus	Section	Subsection	Species	Base pairs (Gb)	Mean coverage	Length	Chloroplast accession	Mean coverage	Targets assembled (%)
*Magnolia*	*Gwillimia*	*Gwillimia*	*M. henryi*	673.65	2,346.05	159,760	OR730771	129.83	239 (100%)
	*Gynopodium*	*Gynopodium*	*M. kachirachirai*	763.59	2,714.55	160,042	OR730772	81.08	239 (100%)
	*Kmeria*	*Kmeria*	*M. septentrionalis*	294.66	813.30	159,838	OR730765	78.24	239 (100%)
	*Macrophylla*		***M. dealbata***	566.90	2,010.76	160,069	OR730743	70.32	239 (100%)
			*M. grandiflora*^a^	1,021.24	NA	161,903	NC_020318^a^	245.68	239 (100%)
			*M. macrophylla*	501.49	1,651.14	160,087	OR730773	85.91	239 (100%)
			***M. rzedowskiana***	396.88	88.06	160,044	OR730717	399.79	239 (100%)
			***M. vovidesii***	504.88	92.58	160,075	OR730707	480.51	239 (100%)
	*Magnolia*		***M. iltisiana***	527.74	71.42	159,672	OR730694	516.45	239 (100%)
			***M. panamensis***	937.28	2,976.42	159,759	OR730755	172.59	239 (100%)
			***M. sharpii***	1,075.06	3,586.13	159,758	OR730758	159.07	239 (100%)
	*Manglietia*	*Manglietia*	*M. grandis*	466.32	1,217.12	160,046	OR730775	132.88	239 (100%)
	*Michelia*	*Michelia*	*M. foveolata*	220.40	473.58	160,070	OR730776	75.52	239 (100%)
	*Rytidospermum*	*Rytidospermum*	*M. obovata*	338.50	917.17	160,087	OR730774	90.59	239 (100%)
	***Talauma***	***Cubenses***	***M. emarginata***^b^	254.80		159,859	OR730766	288.32	239 (100%)
			***M. splendens***	362.46	1,081.98	159,906	OR730761	72.48	239 (100%)
			***M. bankardiorum***	357.37	801.18	159,264	OR730741	125.05	239 (100%)
	***Talauma***	***Dugandiodendron***	***M. coronata***	444.62	53.54	159,223	OR730676	457.59	239 (100%)
			***M. jaenensis***	339.10	65.37	159,277	OR730695	379.65	239 (100%)
			***M. ptaritepuiana***	823.93	77.93	159,188	OR730713	816.14	239 (100%)
	***Talauma***	***Talauma***	***M. allenii***	594.76	91.86	159,879	OR730675	553.81	239 (100%)
		***Talauma***	***M. dodecapetala***	765.72	2,813.31	159,829	OR730744	153.51	239 (100%)
		***Talauma***	***M. kichuana***	275.34	718.39	159,888	OR730749	78.28	239 (100%)
		***Talauma***	***M. lacandonica***	453.55	42.65	159,906	OR730767	458.49	239 (100%)
		***Talauma***	***M. macrocarpa***	79.66	48.92	159,841	OR730698	124.05	239 (100%)
		***Talauma***	***M. mexicana***	457.47	117.34	159,847	OR730700	399.95	239 (100%)
		***Talauma***	***M. ofeliae***	392.61	67.37	159,839	OR730705	380.74	239 (100%)
		***Talauma***	***M. ovata***	476.35	32.76	159,782	OR730768	104.55	239 (100%)
		***Talauma***	***M. pastazaensis***	317.30	910.24	159,811	OR730756	86.97	239 (100%)
		***Talauma***	***M. rimachii***	384.58	66.32	159,829	OR730715	388.68	239 (100%)
		***Talauma***	***M. silvioi***	1,267.35	104.60	159,835	OR730719	1,092.30	239 (100%)
		***Talauma***	***M. virolinensis***	680.10	53.34	159,704	OR730728	650.15	239 (100%)
		***Talauma***	***M. wetteri***	455.86	71.16	159,811	OR730733	432.20	239 (100%)
		***Talauma***	***M. wolfii***	1,034.72	77.38	159,678	OR730734	871.23	239 (100%)
		***Talauma***	***M. zamorana***	631.89	2,222.86	159,758	OR730740	79.68	239 (100%)
		***Talauma***	***M. zoquepopolucae***	486.06	105.34	159,842	OR730763	422.66	239 (100%)
	*Yulania*	*Tulipastrum*	*M. acuminata*	333.53	1,007.07	159,814	OR730769	66.68	239 (100%)
		*Yulania*	*M. biondii*	157.24	416.25	160,014	OR730770	36.41	239 (100%)
*Liriodendron*			*L. tulipifera*^a^	992.91	NA	162,170	DQ899947^a^	90.35	239 (100%)

^a^Chloroplasts of *M. grandiflora* and *L. tulipifera* were downloaded from NCBI GenBank

^b^For* M. emarginata* a nearly complete chloroplast was assembled and used in subsequent analysis. Nuclear targets data can be found in the NCBI Sequence Read Archive BioProject PRJNA1033644. Neotropical sections, subsections and species are in bold

For the nuclear target assembly all the loci selected (239) were recovered for all the species (Table 2). Coverage varied from 36.4 × in *M. biondii* Pamp. To 1092 × in *M. silvioi* (Lozano) Govaerts with a mean of 290 ×. In addition, mean sequence length of each target varied from 341 to 2,758 bp with a global mean of 1,047 bp per target (Table S1). A mean of 250,312 bp was recovered for each individual.

### Phylogenetic analysis and divergence time estimation

**Fig. 2:**
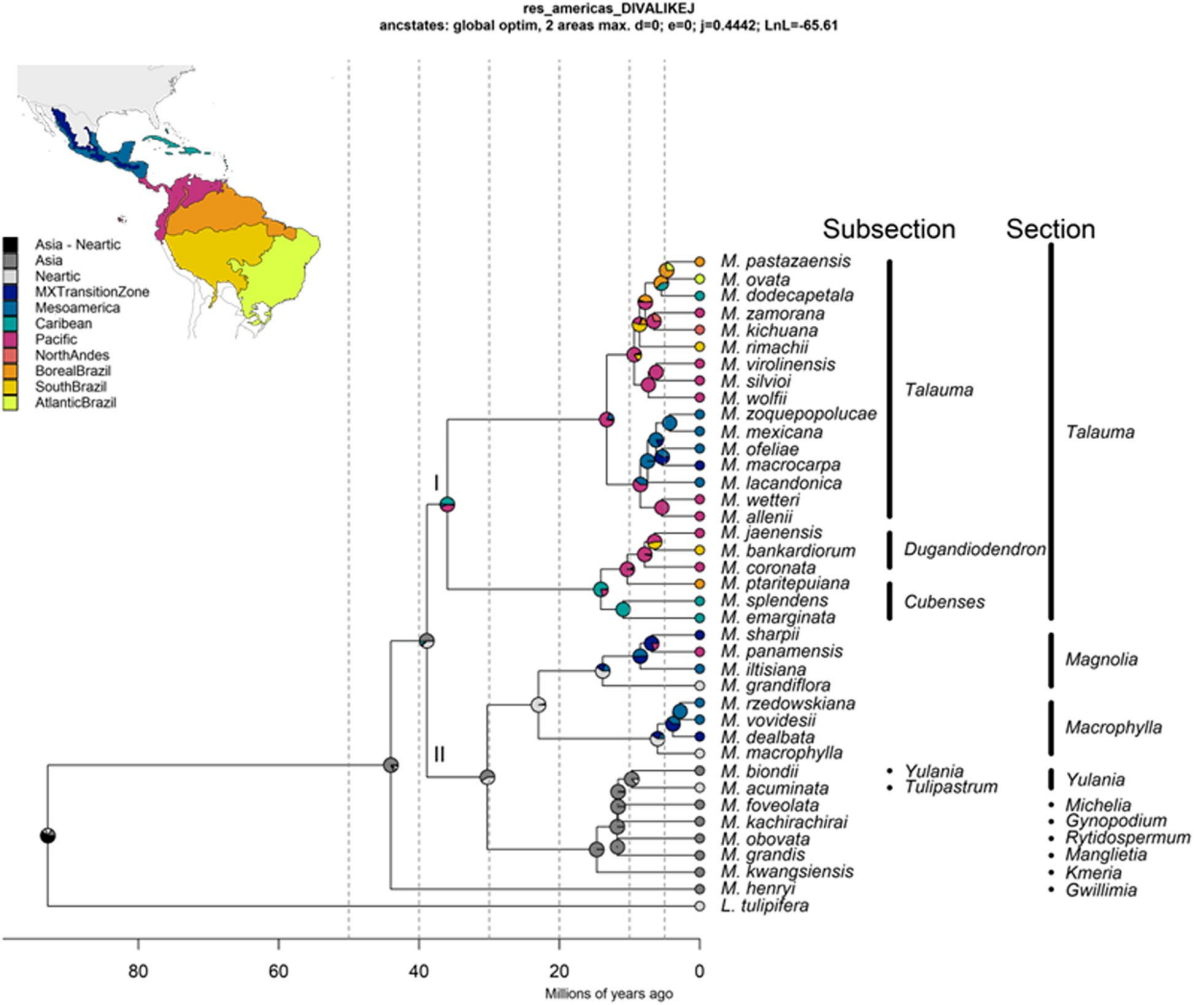
Phylogenomic hypothesis obtained and Ancestral range reconstruction of the 39 Magnoliaceae species. BioGeoBEARS analysis using the “Closing America” DIVALIKE+J model based on the BEAST species tree of the combined 239 nuclear targets and complete chloroplast assembled. Calibration nodes are marked with an *. Pie charts at the nodes represent the probabilities for each of the 10 operational areas. Black color indicates the combination of the Asia and Nearctic regions. Classification follows the one proposed by [36]

The ML trees resulting from the chloroplast dataset resulted in two main clades within *Magnolia*: one including sections *Gwillimia* and *Talauma* and the second with all other sections. Within each clade, monophyletic sections and subsections were recovered. By its part, the analysis of the combined dataset both in IQtree and BEAST revealed that *M. henryi* Dunn from section *Gwillimia* is the sister taxon to all other *Magnolia* species (Fig. 2, Table 3, Appendix 1). The remaining species within the genus formed two main clades: Clade I encompassed the species from section *Talauma*, while clade II consisted of species from the other sections. Posterior probabilities (PP) for these two nodes were the lowest in the analysis (0.75). Most of the other nodes in the results were highly supported either with a PP of 1 or bootstrap values of 1000 (Table 3, Appendix 1). Within section *Talauma*, we identified a clade comprising all species from subsection *Talauma*, and another one including subsections *Cubenses* and *Dugandiodendron*. Clade II comprised all the other sections included in the analysis. Within this, two subclades appeared, the first with sections *Magnolia* and *Macrophylla* and the second with all the other sections: *Kmeria*, *Rythidospermum*, *Manglietia*, *Gynopodium*, *Michelia* and *Yulania* (clade KRMGMY).

**Table 3:** Estimated posterior probabilities (PP) and ages of Magnoliaceae clades according to the BEAST divergence time estimation

		Age		
Clade	PP	mean	min	max
Magnoliaceae^a^	1	92.92	44.39	98
Genus *Magnolia*^a^	1	44.02	44	44.08
Clade I - Clade II	0.75	38.91	37.9	39.85
Clade I (Section *Talauma*)	1	35.97	35.92	36
Subsection *Talauma*	1	13.26	2.99	19.3
Subsections *Cubenses* + *Dugandiodendron*	1	14.08	10.4	16.13
Subsection *Cubenses*	1	10.91	8	12.6
Subsection *Dugandiodendron*	1	10.33	7.34	12.32
Clade II	0.75	30.26	21.15	38.3
Sections *Magnolia* + *Macrophylla*	1	22.98	1.82	34.92
Section *Magnolia*	1	13.83	1.08	20.94
Section *Macrophylla*	1	6.0	0.47	9.01
Clade KRMGMY	1	14.69	0.42	6.74
Section *Yulania*	1	9.7	0.27	24.36

Ages are shown in million years ago and represent the mean, minimum and maximum values for the 95% Highest Posterior Density intervals

Calibrated nodes are marked with an^a^

The divergence time estimation provided insights into the chronological relationships within the Magnoliaceae family. The oldest node identified was the divergence of *Liriodendron* from *Magnolia* (Fig. 2), estimated to have occurred approximately 92.92 mya (Table 3). The second oldest node represented the split between *M. henryi* and the rest of the genus, estimated to have taken place at 44.02 mya. Our results suggest that the separation between clades I and II occurred at 38.90 mya. The subsequent divergence within section *Talauma* happened at 35.97 mya, separating subsection *Talauma* from subsections *Dugandiodendron* and *Cubenses*. Finally, these two subsections diverged from each other approximately 14 mya.

Furthermore, within clade II a subdivision occurred at 30.26 mya, resulting in two distinct subclades. On the one hand, we have the common ancestor of sections *Magnolia* and *Macrophylla*, while on the other hand, the remaining sections diverged. The split between sections *Magnolia* and *Macrophylla* took place at 22.98 mya and the extant species of these section have an estimated age of 13.83 mya and 6 mya, respectively. In addition, the sections from clade KRMGMY experienced subsequent divisions, with the initial divergence estimated to have occurred at 14.69 mya, and this continued until 9.7 mya.

### Ancestral range reconstruction

The results of the ancestral range reconstruction analysis using BioGeoBEARS are provided in Table S2. Among the different models tested, the model “Closing Americas” DIVALIKE+J yielded the lowest AICc value, followed by the “GAARLANDIA” DIVALIKE+J and the Panama DIVALIKE +J models. The best models suggest a shared Asia + Nearctic origin for the family and the *Magnolia* genus. The best models suggest that ancestors of the clades I and II of *Magnolia* first arrived at the Asian region about 44.01 mya (Fig. 2, Appendix 2). From there, ancestors of different clades colonize the Caribbean, Asia, and Mesoamerica at different moments. According to these models, the first colonization occurred about 38.91 mya when the *Talauma* ancestor arrived at the Caribbean (Appendix 2, Table S3). Another migration occurred when the ancestor of the clade KRMGMY split from the ancestor of sections *Magnolia* and *Macrophylla* about 30.26 mya. Finally, two more recent migrations have occurred from the Nearctic into the Mesoamerican and Mexican Transition Regions.

Within the Neotropics, each section exhibited a unique biogeographic history. According to the best models, section *Talauma* originally colonized the Caribbean and later, subsection *Talauma* migrated into the Pacific region. The other clade remained in the Caribbean until the divergence of subsection *Dungadiodendron*, which migrated into the Pacific region.

Within subsection *Talauma* each subclade presented a different biogeographic history. According to the “Closing Americas” DIVALIKE+J, the origin of the clades varied between the Pacific and the Mesoamerican regions (Appendix 2). For sections *Cubenses* and *Dungadiodendron*, all the best models suggest a Caribbean origin for the first and a Pacific one for the second.

Turning to sections *Macrophylla* and *Magnolia*, all the models suggest a Nearctic origin for the ancestor of both clades. Similarly, all the models indicate that a migration into the Mesoamerican and Mexican Transition Zone occurred about 13.83 mya when *M. grandiflora* diverged from the rest of the *Macrophylla* members. By its part, ancestors of the species of section *Magnolia* arrived at the same regions about 6 mya.

## Discussion

### Sequencing and assembly of the Magnoliaceae bait set

Data produced by HTS techniques have proven to be an invaluable tool to address evolutionary questions in different angiosperm lineages [43, 82–84]. They have also been used in Magnoliaceae, both with plastid [38, 85] and nuclear data [43]. In our analysis, the sequencing and assembly of the Magnoliaceae nuclear target dataset developed by Kim et al. (in press) produced excellent results (Table S1). These markers have been used in a parallel study also with significant success [35]. Although the bait kit includes 490 different nuclear markers, the mentioned study found that some of these are not well-recovered, especially in Neotropical species [35]. Because of this, we limited our analysis to only 239 markers. These were recovered by all the species analyzed in this and previous works. This number of markers proved to be sufficient to resolve phylogenetic relationships across all sections of the genus *Magnolia*.

With recent advances in sequencing platforms, there has been an increase in the number of phylogenomic studies utilizing plastid genomes in various angiosperm lineages [43, 86]. This applied to the Magnoliaceae family as well, where studies employing plastid data have provided insights into its evolution, such as the highly conservative nature of the plastome or a classification based on natural groups [38, 85]. However, the combined use of nuclear and plastid sequences is not yet widely implemented [43]. It is known that plastid markers share a common evolutionary history due to their presence in the same cellular unit, and integrating data from different compartments can enhance the resolution of phylogenetic hypotheses [87, 88]. In our study, the integration of both nuclear and plastid data resulted in a robust phylogeny consistent with previously generated knowledge [38, 44].

### Phylogeny of Neotropical *Magnolia*

In general, all the phylogenetic trees resulting from the analysis showed similar relationships. Our results confirm that Neotropical *Magnolia* species belong to three distinct sections: *Macrophylla*, *Magnolia*, and *Talauma*, and exhibit strong support in most clades (Table 3, Appendix 1). However, some discrepancies exist when comparing these results to previous studies. Recent phylogenetic hypotheses support the division of Magnoliaceae into two main clades: one comprising sections *Talauma* and *Gwillimia*, and the second containing the remaining sections [35, 38]. These studies employed either chloroplast data or a combination of chloroplast and nuclear data, utilizing different reconstruction methods such as Maximum Likelihood and Bayesian Inference. Our results from the chloroplast ML analysis follow these relationships between sections *Gwillimia* and *Talauma*; however, results from the nuclear and the combined datasets differ. These analyses place *Gwillimia* as the first diverging section of Magnoliaceae, sister to all other species in the genus (Fig. 2, Appendix 1). This finding aligns with the results reported by [44]. They estimated the divergence times using BEAST and a combination of sequences from eleven genomic regions: five nuclear genes (AGT1, GAI1, LEAFY, PHYA, SQD1), three chloroplast genes (*ndhF*, *rbcL* and *trnK*) and three chloroplast intergenic regions (*atpB-rbcL*, *ndhF-rpl32* and *psbA-trnH*). In their results, they also found that section *Gwillimia* appeared as sister to all other *Magnolia* species [44]. In the case of [43], they performed an analysis combining plastid and nuclear data and found a similar discrepancies between both datasets.

Another difference between the analysis of the different datasets is the relationship between sections *Macrophylla* and *Magnolia*. In the results from the chloroplast dataset, *Macrophylla* is sister to a clade that includes section *Magnolia* and all Asian sections. However, when considering the nuclear and combined datasets both clades appear forming a clade which in turn is sister to the Asian clades. Other studies have found other patterns. In the case of [44], section *Magnolia* resulted sister to a clade that includes *Macrophylla* and another Asian sections (*Tulipastrum*, *Yulania* and *Michelia*). Another study [38] found that *Macrophylla* and *Tuliparia* belong to a clade that is sister to another one that includes *Magnolia* and most of the Asian sections. By its part, [43] found discrepancies between their nuclear and plastid datasets. On the one hand, *Magnolia* and *Macrophylla* were shown to be sister clades, while on the other hand, they found that section *Macrophylla* is sister to a clade that contains sections *Magnolia*, *Manglietia*, *Rytidospermum* and *Oyama*.

Discrepancies between phylogenetic hypotheses are usually attributed to missing data, taxon sampling or the result of an artifact produced by the method used [89]. The relationships of *Gwillimia* found in this work and in [44] do not agree with those in previous studies. This may be due to the limited sampling of *Gwillimia* species in both cases. Similarly, the reduced number of species from sections *Macrophylla* and *Magnolia* usually included in other studies could hinder the resolution in the relationships of these clades. Another aspect to consider is that discrepancies have been reported in the Magnoliaceae when using plastid and nuclear data, which could lead to different topologies [43]. These differences are mainly the position of sections *Magnolia* and *Macrophylla* [35]. The patterns found in different studies for the relationships between sections *Macrophylla* and *Magnolia* suggest that the selections of loci could also be a factor in recovering the evolutionary history of these groups. These results suggest that the evolutionary history told by nuclear sequences could be different from that of the chloroplast sequences.

### Biogeographic history of *Magnolia* in the Neotropics

Section *Talauma* was found to have originated approximately 35.97 mya, making it the oldest Neotropical Magnoliaceae clade (Fig. 2 and Table 3). *Magnolia* and *Macrophylla* have estimated ages of approximately 13.83 mya and 6 mya, respectively. The ages estimated for sections *Talauma* and *Macrophylla* are higher than those reported by [44] for the same clades, with values of 30.3 and 4.8 mya, respectively, while the estimation for section *Magnolia* is lower than the 9.35 mya estimated by them [44]. However, all the highest posterior density intervals overlap. Other studies on the family suggest an origin of between 42 to 35 mya for section *Talauma* and between 32 and 34 mya for the other two sections [13, 42].

Although in general the age estimates from most clades are in line with those reported by previous studies, there are some considerations to keep in mind such as the parameters and the calibration points selected in the analysis. The effect of the molecular clock selected to date a phylogenetic tree has been discussed broadly in different works [70, 90]. It has been stated that the use of a global relaxed clock could produce unprecise dates in the estimations of certain clades [91, 92]. Similarly, the use of different prior models, such as the Calibrated Yule prior or the Birth-Death prior, also influence the estimates of the nodes in the resulting phylogeny, especially for ancient clades that likely present high extinction rates [93]. In the case of Magnoliaceae, the use of a random local clock could benefit the resulting age estimates due to the numerous clades and sections within the family. In contrast, the use of a Calibrated Yule prior could influence the final estimates.

Another aspect that may influence the resulting age estimates of the nodes is the combination of taxa sampled for each clade. It has been discussed that a complete sampling improves both the results of a phylogenetic hypothesis and the precision of the divergence time estimations [94–96]. However, it has been proven in simulations that the effect can be negligible, at least in cases where rate heterogeny is small [94, 96]. In contrast, in empirical datasets the results were not sensitive to incomplete sampling when using multiple calibration nodes [95]. For the case of this study, we used a sampling that includes at least one representative from each *Magnolia* section and the calibration scheme includes two calibration points which resulted in an accurate estimation according to previous studies [42, 44].

The calibration scheme used for the divergence time estimate analysis also could influence the resulting dates of some clades [97]. Other studies focused on Magnoliaceae have used *Archaeanthus* and *Magnolia tiffneyi* as source for the calibration nodes for the family [42, 44]. In our study, the estimated lower limit of the Magnoliaceae node almost overlaps with the maximum value of the *Magnolia* node. Additionally, the interval of dates for the *Magnolia* node is very narrow compared to the rest of the nodes. These results are likely an artifact produced by the calibration schema used in the analysis. A similar effect was observed by Veltjen et al. [44], following a similar calibration. By its part, the calibration of the Magnoliaceae stem node based on *Archaeanthus* is also of consideration. Some authors suggest that the morphological traits place this genus closer to *Liriodendron* in the Liriodendraceae *s. l.* clade [98]. Nonetheless, other studies either suggest the use of *Archaeanthus* as a minimum age constraint for the stem node of Magnoliaceae [99], or do not define a clear position for this taxon [100]. Based on this discrepancy we consider that the use of this taxon as a calibration point for the stem node of the family is appropriate as shown by the results obtained from this and other works [42, 44]. However, future studies in the relationships of *Archaeanthus* could modify the placement of the genus and the estimated dates of the nodes.

The model “Closing Americas” DIVALIKE+J was the best fitting models according to the AICc value. This model included parameters to consider the change in dispersal probabilities between Mesoamerica and the Pacific region in the last 20 mya and the effect of the closure of the Panama land bridge about 3 mya [6, 12]. Other studies have suggested that dispersal rates between North and South America change across time [101], which makes a model that consider these differences more desirable. At the same time, divergence estimates resulted in our analysis suggesting that all the divergences between species occurred before the 3 mya mark. We propose that the “Closing Americas” DIVALIKE+J model presented here is the most appropriate to describe the evolution of the Magnoliaceae in the region because it considers the AICc values, the biogeographic context and the results obtained in our analysis. The discussion presented in the next sections are based on this model.

The ancestral range reconstruction does not definitively identify the origin of the Magnoliaceae. However, the oldest fossils assigned to the family suggest a Western North American origin [39, 40]. From there, the Magnoliaceae could have diverged into the two clades observed in the phylogenetic reconstruction: *Magnolia* and *Liriodendron*. By its part, the genus *Magnolia* shows a higher probability of having an Asiatic origin although it is not decisive. The initial split in this genus occurred around 44 mya, when section *Gwillimia* diverged from the rest of the species. From there, the remaining sections of the genus are divided into two main clades: Clade I and Clade II.

#### The early arrivals: section Talauma

**Fig. 3:**
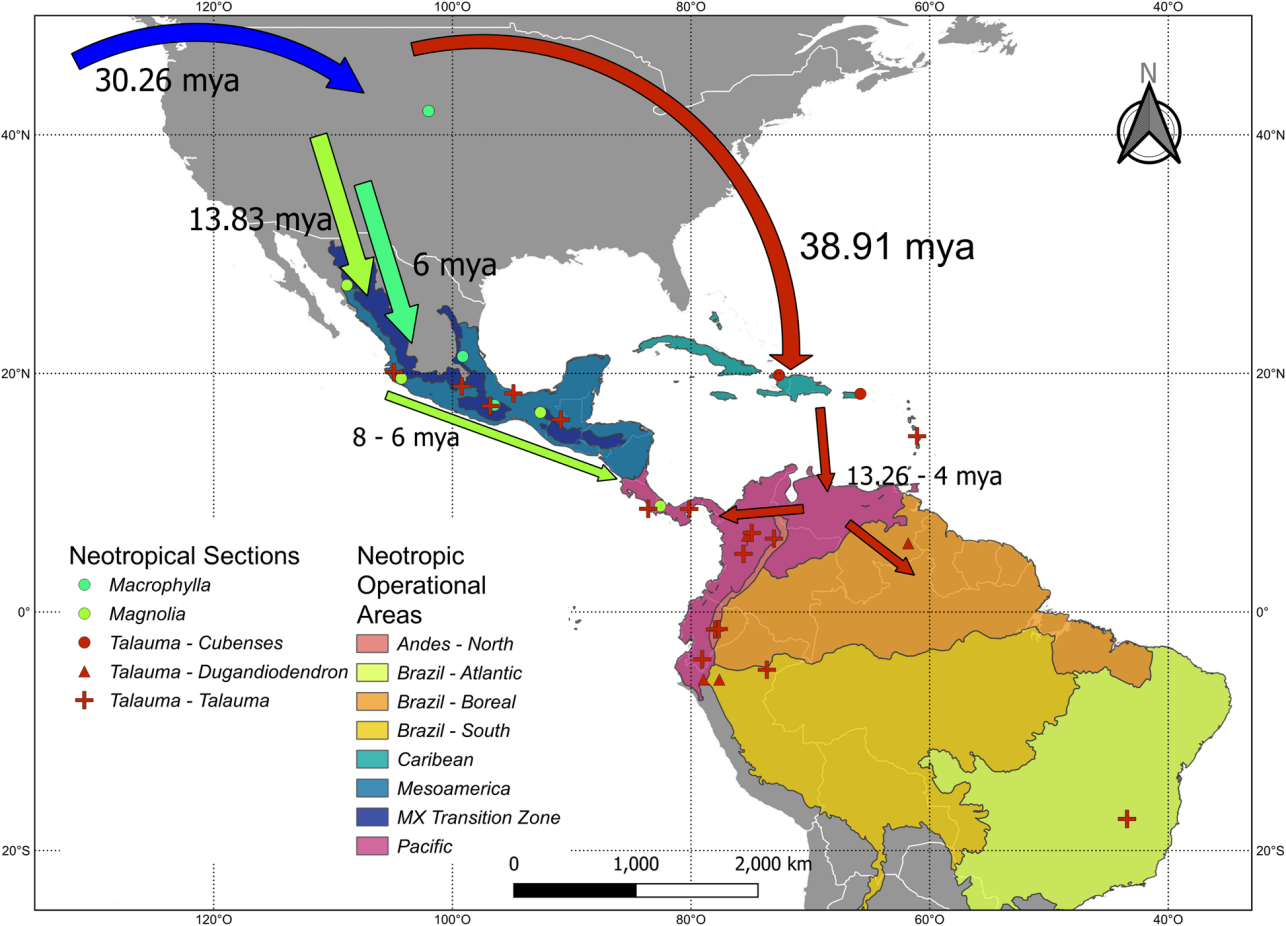
Probable routes followed by the species of the different *Magnolia* sections. Markers indicate the *Magnolia* section to which each species belongs. Colored regions represent the operational areas defined for the ancestral range reconstruction based on the biogeographical provinces of Morrone and Löwenberg-Neto. Blue arrow represents the possible route of Magnoliaceae from Asia. MX stands for Mexican. This figure is an original creation and does not derive from any other source

The colonization of the Neotropics by *Magnolia* species likely began with the arrival of the ancestors of Clade I, corresponding to section *Talauma*, approximately 35.97 mya. This migration probably originated from Asian or Nearctic species that started to radiate southwards into the Caribbean region (Figs. 2 and 3). This colonization pattern has been observed in other tropical plant groups such as *Croton* [102], *Ficus* [103] and *Styrax* [104], as well as in certain animal groups including sloths [105], rodents [106] and amphibians [107]. Benthic foraminifera records suggest a temperature drop of approximately 4 °C around 34 mya [21], which could have transformed the boreotropical regions into a more temperate climate, potentially driving the migration of many angiosperm taxa towards lower latitudes. The migration of biodiversity from the North to South American land masses, prior to the existence of the Panama isthmus, has often been attributed to the emergence of a hypothetical land bridge known as GAARlandia, consisting of a semi-continuous chain of islands extending from current-day Cuba to Venezuela, during the Eocene-Oligocene boundary, approximately 34 mya [20]. This land bridge may have facilitated the migration between North and South America. In the Caribbean and between the continents this may have occurred through island hopping. Another hypothesis for the arrival of section *Talauma* in South America is long-distance dispersal, potentially facilitated by animals or rafting. Long-distance dispersal events have been proposed for several angiosperm lineages that inhabit both North and South America [108, 109]. It has been observed that dispersers of extant Magnoliaceae species usually are birds and large beetles [110, 111] which can transport *Magnolia* seeds over long distances. Although recent studies challenge the validity of the GAARlandia hypothesis [6, 19], our results suggest that the Caribbean could have been a step in the dispersal of *Talauma* species into South America.

#### The temperate followers: sections Macrophylla and Magnolia

While *Talauma* colonized and diversified across the entire Neotropical region, the ancestors of Clade II probably remained in the Nearctic. About 30 mya this clade splits into a group that originates most of the Asian *Magnolia* clades and another including the ancestors of sections *Magnolia* and *Macrophylla*. The Miocene Climatic Optimum, which occurred around 17 mya, is known to have influenced the angiosperm diversity of North America [6]. These climatic factors could have played a role in the dispersal of sections *Macrophylla* and *Magnolia* into the Mesoamerican and the Mexican Transition Zone regions and their subsequent diversification.

The formation of the Panama Land Bridge is considered another event that has shaped Neotropical diversity [112, 113]. Although the Isthmus was fully formed approximately 3.5 mya, the proximity between North America and South America has allowed the movement of many taxa between the two continents, with some migrations occurring even at about 20 mya [22]. Ancestral Area Reconstruction analysis suggests that members of section *Talauma* may have dispersed from Pacific regions into Mesoamerica about 8 mya. At this time, the collision between the North and South American plates had already occurred, and the separation between both landmasses was less than 200 km [22]. This proximity likely facilitated the migration of tropical elements between these. However, for sections *Magnolia* and *Macrophylla*, the closure of the Panama Land Bridge has not allowed the migration between the two continents. This can be attributed to the temperate affinity exhibited by most species in these clades, as the lowlands of the Mesoamerican and Pacific subregions acted as a barrier for taxa from these sections.

Although our results showed the relation between the evolution and diversification of the Magnoliaceae in the region, further biogeographic research on the family is advised to focus on addressing the two remaining unresolved bifurcations considering Magnoliaceae biogeographic history (Table 3). Also, it will be important to address studies focused on more fine-scale biogeographic patterns by adding more species to the phylogenomic trees. Finally, climatic modelling techniques can be used to elucidate which climatic factors play a key role in the current distribution and compare these with simulations of past climatological models to obtain evidence on past distributional hypotheses for the Magnoliaceae.

## Conclusions

Our phylogenetic analyses based on nuclear and chloroplast sequences from 39 Magnoliaceae species give insights into the evolution of the Neotropical species. Results support the monophyly of the American sections *Macrophylla*, *Magnolia*, and *Talauma*. The ancestral area reconstruction suggests that the colonization of the region likely started with the arrival of the boreotropical ancestors of section *Talauma* into the Pacific region of South America about 36 mya. This process could be influenced by the temperature drop of the Eocene/Oligocene transition. Subsequently, this section started to diversify across the entire Neotropical region. The ancestors of the other sections inhabited the Nearctic until at least 14 mya, when the predecessor of sections *Macrophylla* and later *Magnolia* diversified into the Mesoamerican and Mexican Transition Zones. Climatic factors, such as the late Oligocene warming and the Miocene Climatic Optimum likely influenced these dispersal events. Additionally, the proximity between North and South America could have played a significant role in shaping the biogeography of *Magnolia* in the Americas, as it allowed the migration of certain taxa in the last 20 mya, while the lowland tropical regions could act as a barrier for others.

## Supplementary Information

Additional file 1: Table S1. Results from the assembly of the 239 nuclear targets of the 39 Magnoliaceae samples analyzed.

Additional file 2: Table S2. Results from the thirty models included in the BioGeoBEARS analysis.

Additional file 3: Table S3. Range probabilities resulting from the three best models tested in the BioGeoBears analysis.

Additional file 4: Table S4. Bait set used for the analysis.

## Figures and Tables

**Figure:**
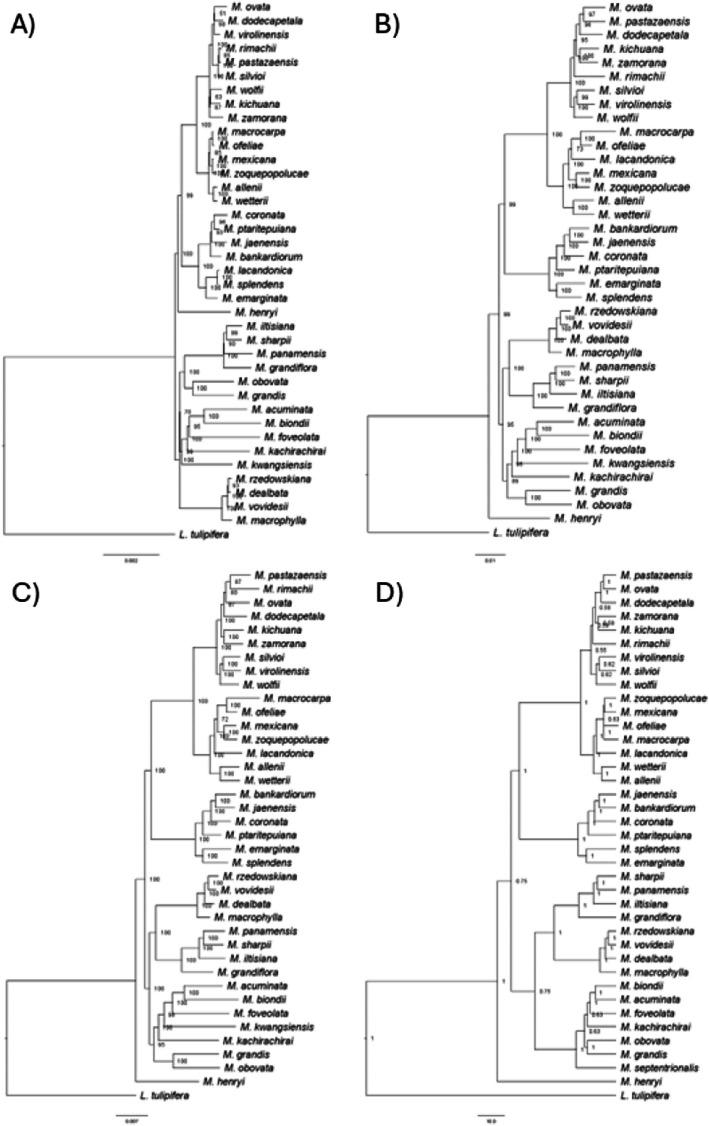
**A** Phylogenetic tree resulting from the Maximum Likelihood analysis of the complete chloroplast dataset performed by IQtree. **B** Phylogenetic tree resulting of the Maximum Likelihood analysis of the partitioned nuclear dataset including 239 nuclear regions performed by IQtree. **C** Phylogenetic tree resulting from the Maximum Likelihood analysis of the partitioned combined dataset including complete chloroplast sequences and 239 nuclear regions performed by IQtree. **D** Phylogenetic tree resulting from the Bayesian Inference analysis of the partitioned combined dataset including complete chloroplast sequences and 239 nuclear regions performed by BEAST

**Fig. 5:**
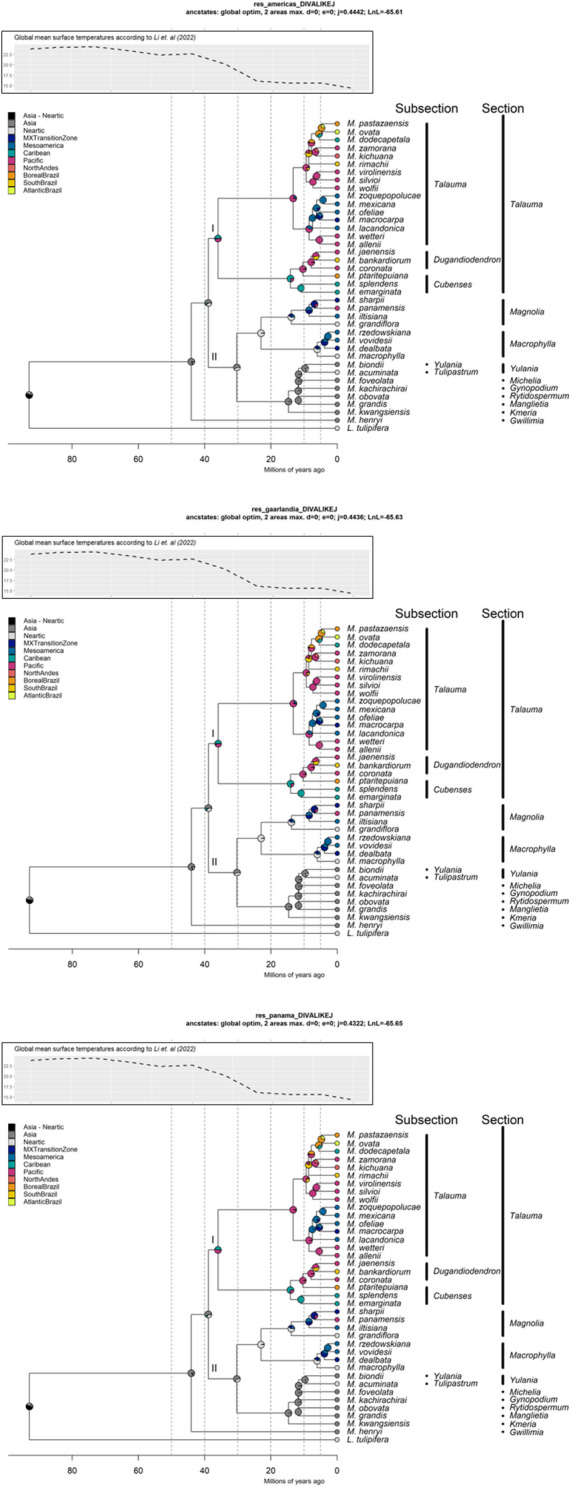
Resulting ancestral range estimations produced by BioGeoBEARS for the three best models according to the AICc values with global mean temperature estimates according to [114]: “Closing Americas” DIVALIKE+J and the “GAARLANDIA” DIVALIKE+J and Panama DIVALIKE+J

## Data Availability

The dataset(s) supporting the conclusions of this article are available in the following resources: chloroplast sequences are deposited in NCBI genbank with accession numbers according to table 2: OR730771, OR730772, OR730765, OR730743, NC_020318, OR730773, OR730717, OR730707, OR730694, OR730755, OR730758, OR730775, OR730776, OR730774, OR730766, OR730761, OR730741, OR730676, OR730695, OR730713, OR730675, OR730744, OR730749, OR730767, OR730698, OR730700, OR730705, OR730768, OR730756, OR730715, OR730719, OR730728, OR730733, OR730734, OR730740, OR730763, OR730769, OR730770, DQ899947. Nuclear baits are included as supplemental material. Raw sequences are deposited in the NCBI Sequence Read Archive (SRA) with the BioProject number PRJNA1033644: SAMN38039693, SAMN38039694,, SAMN38039695, SAMN38039696, SAMN38039697, SAMN38039698, SAMN38039699, SAMN38039700, SAMN38039701, SAMN38039702, SAMN38039703, SAMN38039704, SAMN38039705, SAMN38039706, SAMN38039707, SAMN38039708, SAMN38039709, SAMN38039710, SAMN38039711, SAMN38039712, SAMN38039713, SAMN38039714, SAMN38039715, SAMN38039716, SAMN38039717, SAMN38039718, SAMN38039719, SAMN38039720, SAMN38039721, SAMN38039722, SAMN38039723, SAMN38039724, SAMN38039725, SAMN38039726, SAMN38039727, SAMN38039728, SAMN38039729, SAMN38039730, SAMN38039731. Python, R and shell scrips used in this work can be found in the github repository https://github.com/Zcrass/Magnoliaceae_chloroplast.

## Abbreviations

Mya: Million years ago
HTS: High throughput sequencing
PP: Posterior probabilities
KRMGMY: Clade including sections *Kmeria*, *Rytidospermum*, *Manglietia*, *Gynopodium*, *Michelia*, and *Yulania*
